# Prevalence of Burnout Syndrome and Job Satisfaction in Music Therapists in Spain: A Cross-Sectional, Descriptive Study

**DOI:** 10.3390/ijerph18179108

**Published:** 2021-08-29

**Authors:** Manuel Sequera-Martín, María Isabel Ramos-Fuentes, Elisa María Garrido-Ardila, Carmen Sánchez-Sánchez, Antonia de la Torre-Risquez, Juan Rodríguez-Mansilla

**Affiliations:** 1 Department of Medical-Surgical Therapy, Faculty of Medicine and Health Sciences, Extremadura University, 06006 Badajoz, Spain; manuseqmamt@gmail.com (M.S.-M.); miramos@unex.es (M.I.R.-F.); callas16@gmail.com (A.d.l.T.-R.); jrodman@unex.es (J.R.-M.); 2 Department of Nursing and Physiotherapy, Faculty Nursing and Physiotherapy, Salamanca University, 37007 Salamanca, Spain; csanchez@usal.es

**Keywords:** burnout syndrome, job satisfaction, music therapists, job stress

## Abstract

Background: Burnout syndrome and job satisfaction are topics of increasing interest due to their relevance in people’s health and well-being. Besides, they are considered very relevant in the fields of social and health care studies. Objective: The objective of this study was to analyse the professional profile of music therapists in Spain and the prevalence of burnout syndrome and job satisfaction among them. Methods: This was an observational, descriptive, cross-sectional study, carried out using an ad-hoc online questionnaire, the Maslach Scale and the general satisfaction scale on a sample of employed Spanish music therapists with more than two years of working experience in Spain. Results: Eighty questionnaires were analysed. The majority of the participants were between 30–39 years old (38.8%) and were women (85%). They combined their profession with other occupations (76.3%), mostly in care roles with a fix term contract and were self-employed (40%). The prevalence of burnout syndrome was 3.8% (*p* < 0.001) and the predisposition or tendency to develop this condition was over 57.5% (*p* < 0.001). The highest levels of burnout syndrome were found in professionals with trainee contracts (*p* = 0.001), in those who were providing training (*p* = 0.021), who attended 6 to 10 patients per week (*p* = 0.001), who were usually working with a therapist colleague (*p* = 0.046) and those who did not take prescribed psychotropic drugs (*p* = 0.034). The highest level of job satisfaction was observed in music therapists working in the field of disability (*p* = 0.010) and mental health (*p* = 0.022) and with seniority in their job position. The lowest level of job satisfaction was seen in music therapists with trainee contracts (*p* = 0.041), with less working hours per week (*p* = 0.016), working in the field of education (*p* = 0.006) and in those who did not feel valued by their colleagues (*p* < 0.001) or by the director of the centre where they worked (*p* < 0.001). Conclusions: Based on the results of this study, Spanish music therapists show a low prevalence of burnout syndrome but a moderate-high predisposition to develop it. Music therapists with burnout syndrome are those who work longer hours and perform their job in palliative care setting. In general, music therapists have a high level of both intrinsic and extrinsic job satisfaction. The lowest level of job satisfaction was found in music therapists with trainee contracts and the highest in music therapists with senior positions.

## 1. Introduction

The World Health Organisation (WHO) [1] and the International Labour Organisation (ILO) [2] reports and recommendations on health, care for professional-workers and the prevention of risks associated with work, state that the work environment can be the cause of numerous occupational pathologies.

In Spain, the prevention of psychosocial risks and work-related stress has been studied in areas such as healthcare and education [3,4,5]. Socio-health professionals are subject to the negative effects of stress and its consequences for their health, due to factors like work shifts, the hierarchy of the working systems or to the fact of providing services to vulnerable people. It is in this context, that the concept of burnout syndrome emerged with a growing interest from health professionals, although it has been associated with psychological and occupational health and has been widely studied for several decades [6].

The scientific evidence indicates that most health and social care professionals working in direct contact with patients [7,8] suffer from emotional exhaustion, depersonalisation and lack of personal fulfilment in their work environment [7,8,9]. According to Kearney et al. [9] Burnout syndrome is associated with suboptimal patient care practice, malpractices of the professionals and lower dissatisfaction. Christina Maslach defines burnout syndrome as ‘chronic stress produced by contact with customers that leads to exhaustion and emotional distancing from customers at work’ [6].

In addition, experts in the field indicate that it is important to know the degree of burnout syndrome of people at work in order to plan the appropriate and optimal social and health care management [10]. The satisfaction variables include those related to the job itself, the working hours, remuneration, the environment and performance, the possibility of professional development and promotion, the salary, satisfaction and motivation [11]. In particular, job satisfaction is defined as the pleasure that someone gets from being able to do his or her job properly, as well as the satisfying feeling of contributing to the organisation, colleagues and society [12].

Music therapy is a discipline that although it has a long history as a socio-health profession in European countries, in Canada and in the United States [13], it is relatively young in Spain. In this country, in order to become a music therapist, you need to take a Master’s Degree in Music Therapy of at least 60 ECTS (European Credit Transfer and Accumulation System). This training can be completed in both public and private universities as well as in some private training centres. In other countries such as Argentina, there is degree in Music Therapy that involves four to five years of education, depending on the university [14].

A music therapist must be trained in clinical, musical and music therapy areas. Training in the clinical field should include knowledge of human physiology, pathology, psychology and psychopathology, among others. In relation to the musical area, the music therapist must acquire knowledge of the basic theory of music, should play musical instruments such as the guitar, the piano or singing and must have knowledge of composition and musical improvisation. Regarding the music therapy area, they must be trained in how to create music adaptations to achieve the therapeutic objectives established in the treatment plan. They must also be able to assess, plan, implement and report the music therapy interventions [15].

Another important part of the training in music therapy is the process of self-experience, in which the student experiences in first person a personal therapeutic didactic process in music therapy. This is essential to be able to practice the profession in optimal conditions [16].

Regarding professional opportunities, although music therapy in Spain is a profession in the process of development and professional regulation, there are both public and private programmes and projects that are being carried out by qualified music therapists in areas such as geriatrics, disability, mental health, hospitals and palliative care [17].

Music therapists are exposed to stressful situations when working with people at risk of vulnerability and in conditions that are not always ideal. Therefore, they have predisposing factors to suffer burnout syndrome just like other professions that work in direct contact with people who have a disease or medical condition [18,19,20,21,22,23,24,25,26,27,28,29].

Different research studies conducted in different countries have evidenced the need for self-care and prevention of burnout syndrome in music therapists in Canada [20] or the presence of moderate job satisfaction among British music therapists [25]. Hills et al. [21] highlighted the importance of teamwork for music therapists in their study conducted in the UK. In addition, the scientific evidence has shown differences within countries in relation to this syndrome. While research conducted in Korea showed a high level of burnout syndrome in music therapists [19], the results of the studies carried out in UK [21], Argentina and Brazil [22,30] showed that music therapists had a lower level of this syndrome when they were working within a team.

In 1987, the first studies related to burnout syndrome in music therapists were published. The study by Oppenheim [18] can be highlighted as this author found that only 4% of American music therapists had a moderate level of this syndrome. Subsequent studies such as that of Kim [19] assessed the relation between job satisfaction, self-esteem and burnout in Korean music therapists. Their results showed an inverse relationship between job satisfaction and burnout syndrome. Other studies conducted by Carvallo [22] or Dos Santos [30] approached burnout syndrome in professionals and music therapists in Argentina and Brazil, respectively. They concluded that music therapy can be an ideal strategy for the treatment of the negative effects of stress and burnout syndrome.

The scientific evidence has also identified a relationship between quality of life, emotional exhaustion and self-esteem [31]. Diverse investigations to explore the issue of job satisfaction in more depth have been carried out in different countries such as the United Kingdom [25], the United States [26,27,29] or Argentina [22]. Stewart [25] carried out an analysis of the state of music therapy in the United Kingdom and found that the majority of music therapists worked part-time and were moderately satisfied. Braswell [29] conducted another study in the United States to analyse the job satisfaction of music therapists registered with the American Music Therapy Association. The results showed that those professionals who had higher remuneration and better quality of life also had higher job satisfaction levels and were more emotionally stable to perform their work under optimal conditions. Besides, Kim [19] studied music therapists’ job satisfaction, self-esteem and professional and emotional exhaustion in a descriptive study of American music therapists. The results predicted an inversely proportional correlation between job satisfaction and the level of burnout.

Despite all the scientific research conducted internationally, no studies that analyse job satisfaction and burnout syndrome in music therapists in Spain has been found in the literature. Therefore, the aim of this study was to analyse the professional profile and the prevalence of burnout syndrome and job satisfaction in Spanish music therapists.

## 2. Materials and Methods

This study was a descriptive, analytical and cross-sectional study that was approved by the Bioethics and Biosafety Commission of the University of Extremadura on the 8 October 2019 (registration number 216/2019).

### 2.1. Characteristics of the Sample

The sample consisted of music therapists working in Spain at the time of participation in the study and who were working in the social-health or educational field in both public and private centres.

The inclusion criteria were: to be a qualified Music Therapist with a Music Therapy degree certified by the EMTC (European Music Therapy Confederation) with at least 60 credits from the European Credit Transfer System (ECTS) or to have completed a degree in Music Therapy in Argentina; to be employed during the study; to be 18 years of age or over; to have more than 2 years of work experience as a music therapist; acceptance of the professional music therapist to participate in the study and to sign the informed consent form.

The exclusion criteria were as follows: music therapists or music therapy students who did not meet the above criteria; music therapists who were on sick leave at the beginning of the study; professionals with less than two years of clinical experience working as music therapists; those music therapists who only performed administrative work, internships, training or voluntary work with no contractual relationship with patients/users.

### 2.2. Procedure and Data Collection

The research was conducted by means of the convenience sampling method. Music therapists belonging to professional associations of music therapists throughout Spain were contacted via email. A total of 180 invitations to participate in the research were sent. All the professionals were informed that their participation in the study was anonymous and voluntary and that no incentives would be offered. Together with the link to complete the questionnaire and the scales, all the information about the study, the use and protection of the data and informed consent to participate in the research (obligatory to complete in the case of participation) were enclosed. The participants could access the questionnaire and scales online and the replies were downloaded and collected in an Excel sheet. Data were stored in an encrypted computer and only the authors had access to the information during all stages of the study.

The questionnaires were available from March to May 2019, after which time data collection was completed. The data were processed anonymously, eliminating the personal data of the respondents.

### 2.3. Assessment Tools

An ad-hoc questionnaire was use to collect the data. The design of the questionnaire was based on the most recent literature on the topic of the study [32,33,34]. The questionnaire consisted of 41 items which included: sociodemographic data (gender, age, marital status, place of work, area of intervention which could be in the field of disability, geriatric, hospital, etc.) and variables related to occupational and personal aspects. These variables reflected the working conditions of music therapists and included the number of working hours as a music therapist, the type of work they were doing and whether they felt valued by their employers, by their colleagues, by the relatives of their patients or by their patients themselves.

The following scales were also used:

The Maslach Burnout Syndrome Inventory-Human Services Survey (MBI-HSS). Published by Maslach and Jackson in 1986, it is the most widely used and most researched questionnaire for measuring job burnout syndrome (WBS) [35]. It consists of 22 items that are rated on a Likert-type scale indicating the level of agreement or disagreement with a statement or items. Different adjectives are rated on a scale ranging from 0 (never) to 7 (every day). This inventory is divided into three subscales (emotional exhaustion, depersonalisation and self-fulfilment). The scores obtained can be rated as low, medium or high. The total score of the scale will be the result of the total sum of each of subscale. According to the experts, the Spanish adapted version of this questionnaire is considered valid and reliable for the assessment of burnout syndrome [36].

The General Satisfaction Scale. This scale was developed by the authors Warr and Wall in 1979 [24,37]. It is composed of 15 questions that collect three types of information related to job satisfaction [24]: general, extrinsic or intrinsic satisfaction. The scores vary between 15 (minimum satisfaction) and 105 (highest satisfaction), so that intrinsic satisfaction is between 7 and 49 points and extrinsic satisfaction between 8 and 56. An exploratory factor analysis of Warr’s General Satisfaction Scale was carried out using the principal axes method. The results showed that the scale is valid and reliable in its Spanish version [24].

### 2.4. Statistical Analysis

A descriptive analysis of the data was carried out. It included the absolute and relative (%) distributions for categorical variables, and the minimum, maximum, mean and standard deviation for continuous variables. Continuous variables were tested for normality of distribution using the Kolmogorov–Smirnov tests. Bivariate analyses were performed to analyse the relationships between the level of burnout syndrome and the level of satisfaction with the rest of the socio-occupational and psycho-occupational factors. Given the nature of the variables, and in accordance with the results of the Kolmogorov–Smirnov test, the differences by groups were analysed using the t-Student tests (in the case of 2 groups), with the Levene’s test (for equality of variances), or with an ANOVA (in the case of 3 or more groups). Finally, the prevalence of ‘confirmed burnout syndrome’ or ‘evidence of burnout syndrome’ was determined by point estimates and 95% confidence intervals. Analyses were carried out with the statistical package IBM SPSS Statistics v.24. Observed differences were considered statistically significant for values of *p* < 0.05.

## 3. Results

Of the 180 questionnaires sent, 80 were completed by the music therapists and analysed. The majority of the participants were women (85%) aged between 30 and 49 years (67.6%), with a Master’s Degree in Music Therapy (81.3%) and with stable partners (51.3%). The socio-demographic variables can be seen in Supplemental Table S1.

### 3.1. Socio-Occupational Characteristics of the Sample

The socio-occupational characteristics of the sample are shown in Supplemental Tables S2 and S3. Most of the participants (76.3%) combined their job as music therapists with other occupations. Most of them (60%) had a job which involved the care of patients and 40% had permanent or indefinite contracts and were self-employed. 62.6% had been working as music therapists for less than 4 years as music therapists, 62.5% had their current job for less than 4 years (and 62.5%) were working in private centres.

The results also indicated that many music therapists were working short hour-shifts (36.3%) mainly in the evenings (Table S2). In addition, half of the professionals provided training. The most frequent fields of work were related to disability and education. It is noteworthy that just over half of the participants (55%) combined music therapy with another job on a regular basis and that a quarter of the sample had the intention of leaving their job in the last year and were looking for a new one.

In 30% of the participants of the study, the enthusiasm for their job had diminished. All workers felt valued by patients and relatives, but some did not feel valued by their colleagues (3.8%) or by the director of the centre (19%) (Table S3).

### 3.2. Characteristics of the Occupational and Psychosocial Health of the Sample

Regarding the occupational health characteristics of the participants (Table 1), it is interesting to highlight that 17.5% of the music therapists suffered from a chronic medical condition. Besides, 73.8% of the respondents carried out some activity to disconnect from work. The main actions carried out in the last month were of a social nature, such as meeting with family (71.3%) and friends (67.5%). It is also worth noting that 65% of respondents went on short holidays.

The psycho-occupational health of the sample can be seen in Table 2. The mean level of emotional fatigue was 23.44 (SD = 12.55) while 37.5% of the sample showed a high level of emotional exhaustion. Although 37.5% of the participants showed a low level of personal accomplishment, the level of depersonalisation was low (92.5%), with a mean of 2.11 (SD = 3.25). The mean level of burnout syndrome as a whole was 63.44 (SD = 13.88), although only 3.8% had ‘confirmed burnout syndrome’ (including high emotional exhaustion, high depersonalisation and low personal accomplishment). In addition, 57.7% had ‘indications of burnout syndrome’, fulfilling at least one of the above conditions. On the other hand, the mean level of satisfaction was 73.59 (SD = 19.69), with a slightly higher level of extrinsic satisfaction compared to intrinsic satisfaction.

Supplemental Table S4 shows the results of the normality tests. Except for the subscales of emotional exhaustion and depersonalisation, the rest of the scales/subscales followed a normal distribution according to the results of the Kolmogorov–Smirnov test.

The relationships between socio-occupational and psycho-social factors with burnout syndrome and the level of job satisfaction can be seen in Supplemental Tables S2 and S3 and in Table 1.

The main group differences of burnout syndrome included higher levels in those music therapists with a trainee contract, those who provide training, those who treat between six and ten patients per week, those who usually work with a therapeutic partner or colleague and those who do not take psychotropic drugs prescribed by a doctor. It is also noteworthy, the fact that there was a higher level of burnout syndrome in palliative care workers and in people who did not attend training. However, these data were not statistically significant.

In terms of occupational health characteristics, a higher level of burnout syndrome was observed in people who had not carried out music therapy in the last month and who went on specialised retreats. The analysis showed that the level of burnout syndrome depends on the frequency of these activities. Other relationships, which were not statistically significant, suggested that there is a relationship between a higher level of burnout syndrome and doing activities such as yoga or self-care activities.

On the other hand, the lowest level of job satisfaction was associated with people with internship contracts, with fewer working hours per week, who combined music therapy with other jobs, who worked in the educational field, who were less enthusiastic about their job, who had tried to leave their job, as well as those who did not feel valued by their colleagues or by the director of the centre. In contrast, the level of job satisfaction was higher among those working in the disability and mental health fields.

It was also observed that job satisfaction was higher the longer the music therapist had been in the job, in people working in a hospital setting, in those who had not started looking for another job in the last year and in those who were not taking medically prescribed psychotropic drugs. However, these results were not statistically significant. Besides, the level of job satisfaction was higher in people with chronic medical conditions, in those who carried out music therapy activities and in people who organised meetings with friends. In contrast, it was lower in people who carried out activities to disconnect from work.

The results indicated that the prevalence of confirmed burnout syndrome in the sample was 3.8% (simultaneous presence of high emotional exhaustion, high depersonalisation and low personal accomplishment). Nevertheless, the prevalence varied in the sample between 0.8% and 10.64% with 95% confidence. The prevalence of signs of burnout syndrome (understood as the presence of any of the three symptoms) was higher (57.5% in the study sample), and could vary between 45.94% and 68.49%, with 95% confidence. The data related to the prevalence of the burnout syndrome can be found in Table 3.

The correlation between burnout syndrome and job satisfaction is shown in Table 4. There is an inverse correlation between the different variables of the Maslach burnout Syndrome Inventory and the different variables of job satisfaction. The correlations of the variables emotional exhaustion and personal accomplishment with job satisfaction are more significant than the other variables.

## 4. Discussion

The present study provides clear evidence of the situation of the music therapy profession in Spain. It reveals that, despite the fact that Spanish music therapists show signs of burnout syndrome, only a low percentage actually suffer from it.

As other research has observed [21,38,39,40], music therapy is predominantly female (85%). This profession, as in most social-health and educational professions, is studied and practised mainly by women [38,39,40,41,42,43,44,45]. Generally, women possess qualities of caregiving tasks such as empathy, patience or detecting and attending other persons’ needs and have traditionally occupied professions related to care, teaching or health [44].

In terms of seniority, our study indicates that only 6.3% had long working experience as a music therapist (between 10–29 years). We consider that this could be mainly because in Spain, this discipline is a relatively young profession and 55% of music therapists combine this work with other professional activity on a regular basis. This data differs from countries such as the United States, where most professionals are dedicated only to their work as music therapists [27]. In other countries, for example Argentina, music therapy is regulated as a social and healthcare profession and is also integrated into the public health system [22]. In our sample, the majority of participating music therapists work in education (48%) or healthcare (mainly in disability and 45% in geriatrics).

Job satisfaction is a key indicator for understanding the state of the music therapy profession in Spain. Qualified, motivated and satisfied professionals will have a better quality of life at work and, therefore, will have less stress and burnout syndrome. Our study sample showed a very acceptable job satisfaction (73.56) which coincides with other research on music therapists from America [27] or from the United States [30]. In contrast, other studies carried out on English [25] or Korean [13] music therapists showed higher job satisfaction than the data provided in our research. However, the level of job satisfaction, according to Rainey [46] is determined by several elements: job design, personal characteristics, salary, promotion, job security, supervision, work group characteristics, participation and organisational structure and work environment. Therefore, it is necessary to make the necessary changes in the work environment and in the social perception of the music therapist’s work.

In general, it can be assumed that music therapists like the work they do. It is a vocational profession and although the working conditions could be improved, such as salary, working hours or professional recognition, they feel satisfied and are proud to do their job [19,25,29].

In our research, the lowest job satisfaction was found among music therapists with internship contracts, who had few hours of work per week and professionals who had no enthusiasm for their current job. The highest job satisfaction was found among music therapists working in mental health and disability and those with more years of seniority in the job. These results coincide with those of the study conducted by Goodging [27] in which he showed that the more years of seniority as a music therapist, the higher the job satisfaction.

With regard to enthusiasm and motivation for the job, Decuir’s study [28] evidenced that music therapists who were less enthusiastic or less motivated in their job had less job satisfaction, data that is in line with our results. This may be because an unmotivated music therapist will try to change jobs, look for other work alternatives, and will not have much involvement with both patients and the institution where he or she works.

The prevalence of burnout syndrome gives us an insight into the situation of music therapists who work with patients and how many of them are in a situation of serious vulnerability. Carvallo [22], in his research on burnout syndrome in Argentinian music therapists, determined that there was no burnout syndrome among the participants, although some had signs of suffering from it. In our sample, the prevalence of confirmed cases of burnout syndrome was 3.8%, a result that does not coincide with the results of Fowler [38] who found a prevalence of 10%. However, our data is closer to the results reported in Berry’s study [47], which showed a prevalence of confirmed burnout syndrome of 5% among the study sample.

The studies available in the scientific literature that analyse burnout syndrome in music therapists [19,22,38,47] indicate that, although they suffer from emotional exhaustion, the variables of depersonalisation and personal accomplishment do not stand out as music therapists do not suffer from depersonalisation, nor do they have low personal fulfilment. This constitutes a very positive finding in the profession of music therapists which coincidentally has the objective of trying to humanise health.

In our research, we did not observe emotional exhaustion or depersonalisation. These data coincide with the results of other published studies [21,38], in particular, with those carried out on music therapists from the United States [39]. This may be due to the fact that music therapists receive training that raise awareness on the importance of the emotional care of future professionals and provide tools to perform the necessary supervision and self-care measures. Training and tools can help professionals to avoid the possible negative repercussions that can appear when working both with patients or with healthy subjects.

Spanish music therapists, in general terms, have an acceptable personal acomplishment (37.3%) in the roles and responsibilities that they perform and in the jobs they occupy, which is in line with similar results obtained in other studies [21,38,47]. However, our figures differ from the low personal accomplishment of Korean music therapists (20.96%) [19]. Besides, we observed a direct or positive relationship between the Maslach Burnout Inventory (MBI) and the emotional exhaustion and depersonalisation. This indicates that people who have high burnout syndrome will also have high scores of emotional exhaustion and depersonalisation. This is consistent with the data provided by Kim [19] and Berry [47] in their research in music therapists and with the studies of Butler [45] and Yu [48] conducted in teachers. Therefore, the low job satisfaction could be considered as a possible indicator of burnout syndrome among music therapists.

In our research, music therapists with the highest levels of burnout syndrome were those with trainee contracts, those who provided training, and music therapists who treated between six and ten patients per week. In this respect, we have not found in the literature any studies that analyse the relationship between burnout syndrome and the type of work contract that music therapists have. However, a study conducted by Galicia [43] studied the relationship between the type of contract and the degree of stress in teachers in Mexico. This study concluded that the more hours per week these professionals worked, the higher the level of stress they had. These results do not coincide with those obtained in our study, as music therapists who showed the highest level of burnout syndrome were music therapists employed as trainees who had a training period associated with the contract. This may be due to the fact that only a small percentage of Spanish music therapists who participated in our study worked exclusively as music therapists and the rest had to combine their job with other professional activities [40].

We consider that a music therapist who works full time has more job and personal stability. For example, he or she will have more peace of mind than a music therapist who works only a few hours in this profession and has to move from one centre to another in order to carry out music therapy sessions on an itinerant basis. Regarding the relationship with the number of hours per week working as a music therapist, we have not found in the literature any study that supports our results. Nevertheless, it is important to highlight that in Spain, music therapists do not work many hours a week. This may be due to several factors: there is possibly a low social demand for music therapy treatments at present, there is no regulation of the profession at a labour and legislative level, there are no public positions for music therapists in social-health and/or educational centres and many of the music therapy initiatives and projects in Spain are implemented in the private sector.

In order to develop professionally and to be able to survive financially, music therapists must combine their work as music therapists with other professional activities (psychology, music teachers, secondary school or conservatory teachers or occupational therapy among others). This implies that music therapists work very few hours per week as professional music therapists. We would like to think that the regulation of the profession would bring a great improvement to the development and implementation of music therapy in the social-health and/or educational system, as has occurred in other countries such as Italy, Switzerland, Denmark, Germany or the United Kingdom [46,49,50,51].

In relation to the level of work-related stress of our sample, the highest level of burnout syndrome was observed in music therapists working in palliative care. This can be understandable as the emotional strain and the type of patients they care for can have a negative impact on the professionals. On the contrary, Carvallo and Ilariucci [22] demonstrated that there was a higher level of work-related stress in music therapists working in public education and the penal system. In Spain, music therapy in public education and in the prison system is rarely implemented, and therefore, Spanish music therapists tend not to have experience in these fields.

Our results showed that, regarding the relationship between burnout syndrome and self-care activities, our sample had a tendency to carry out some self-care activities (83.88%). However, most of them were performed once or twice a week (34.6%). These figures are similar to music therapists from United States who, despite being more aware of the importance of these activities than the Spaniards (37%), also carry out self-care activities once or twice a week [47].

We have observed that music therapists with higher levels of burnout syndrome and job satisfaction did some kind of self-care activity or yoga. However, the only study on music therapists that we have found in this respect is that published by Berry [47]. He concluded that there is an inverse relationship between greater self-care and less depersonalisation and a direct relationship between self-care and personal accomplishment. These results do not coincide with ours and may be due to the fact that Spanish music therapists, as a consequence of the burnout syndrome they suffer, seek self-care and yoga activities to reduce it. A music therapist who is burnt out with his or her work will tend to do self-care activities and disconnect from that professional activity.

On the other hand, music therapists who have lower job satisfaction are those who perform some activities to disconnect from work. Berry [47] stated that those music therapists who carry out some self-care activities per week have greater job satisfaction, which does not coincide with our results. This may be due to the fact that most music therapists in Spain combine their job with other employment and sometimes they do not have time to carry out any other activities apart from music therapy.

No other relevant studies on music therapists in terms of whether they do any activity to disconnect from work have been found in the scientific literature. However, there is similar research published on social and healthcare professionals such as the work of Méndez-Campos [41]. This study showed that social and healthcare professionals (doctors, nurses, assistants, physiotherapists and occupational therapists) working in residential homes for older adults carried out activities to disconnect from work like meeting with family or friends (79.1%) or travelling (43.1%). In our study, 73.8% of music therapists carried out some kind of social activity to disconnect from work, including visiting family members (71.3%), meeting with friends (67.5%) or travelling (65%). The comparison of the data shows higher percentages in our study than in the study by Méndez-Campos [41].

An inverse or negative correlation was observed between the MBI and the Total General Satisfaction Scale and therefore, extrinsic and intrinsic satisfaction. Hence, the participants who had a low MBI value will have high values of job satisfaction. Consequently, the higher the job satisfaction of a music therapist, the lower the level of burnout syndrome he/she will present, as job dissatisfaction can lead to burnout syndrome.

### Limitations of the Study

Cross-sectional studies, also known as prevalence studies, aim to study both exposure to a given cause or to a disease and the presence of that cause or disease in a specific, well-defined population at a given time. This type of study does not allow to know the evolution over time, what happened before and after the time at which the measurement was made [49], or as in our case, whether exposure to stressors preceded the burnout syndrome or vice versa. However, the information that is obtained from this type of study is very useful for assessing the health status of a community and for determining its needs.

The study was initially prepared for approximately 200 respondents, of which 80 were eligible to participate and completed the survey. This level of participation may have been due to the fact that the questionnaire may be rather large, with attached scales, and perhaps not all participants had the time to complete it or they did not complete it because they felt that the procedure could be lengthy. However, we must emphasise that participation was comparable to similar studies such as those of Berry [47], Kim [19], Carvallo and Ilariucci [22] or Decuir and Policastro-Vega [28]. Besides, the specificity of this population and the small number of qualified music therapists in Spain makes this sample representative of Spanish music therapists.

On the other hand, Maslach [6] also claims that people living in Europe are less accustomed to participate in studies, surveys or extensive research. The authors consider that this may be because the public expression of some emotional aspects of the burnout syndrome may be socially better viewed at the individual level in the society of the United States than in Europe, where feelings of group solidarity appear more significantly.

## 5. Conclusions

Spanish music therapists present a low prevalence of burnout syndrome but a moderate-high predisposition to develop it. Spanish music therapists who present burnout syndrome are those who work the longest hours and in the palliative care setting. In general, they have a high level of both intrinsic and extrinsic job satisfaction. The lowest level of job satisfaction was found in music therapists with internship contracts and the highest in music therapists with seniority. There was an inverse relationship between burnout syndrome and job satisfaction, the higher the level of burnout, the lower the job satisfaction.

## Figures and Tables

**Table 1:** Occupational health characteristics of the sample and the relationship with the Maslach’s scores and the level of job satisfaction.

Outcome	Category/Option	*n*	%	Maslach Mean Score (SD)	*p*-Value	Mean Job Satisfaction (SD)	*p*-Value
Suffering from a chronic illness	Yes	14	17.5	60.57 (15.49)		82.07 (13.32)	0.025 ^c^
	No	66	82.5	64.05 (13.57)	0.398 ^a^	71.79 (20.41)	
Activities in the last month. Relaxation	No	51	63.8	64.14 (15.06)		71.37 (19.68)	0.184 ^a^
	Yes	29	36.3	62.21 (11.66)	0.525 ^c^	77.48 (19.42)	
Activities in the last month. Music therapy	No	61	76.3	66.66 (13.62)	<0.001 ^c^	70.2 (19.86)	0.005 ^a^
	Yes	19	23.8	53.11 (8.9)		84.47 (14.9)	
Activities in the last month. Yoga	No	60	75	61.9 (13.9)	0.086 ^a^	72.9 (20.44)	0.592 ^a^
	Yes	20	25	68.05 (13.09)		75.65 (17.54)	
Activities in the last month. Mindfulness	No	62	77.5	61.79 (12.5)	0.104 ^c^	74.81 (18.43)	0.307 ^a^
	Yes	18	22.5	69.11 (17.05)		69.39 (23.63)	
Activities in the last month. Mini-meditation	No	69	86.3	62.32 (13.12)	0.071 ^a^	72.71 (20.17)	0.321 ^a^
	Yes	11	13.8	70.45 (17.01)		79.09 (16.03)	
Activities in the last month. Identification of emotions	No	66	82.5	63.45 (14.31)	0.981 ^a^	72.74 (19.43)	0.408 ^a^
	Yes	14	17.5	63.36 (12.1)		77.57 (21.14)	
Activities in the last month. Emotional management	No	73	91.3	63.07 (14.16)	0.446 ^a^	73.52 (19.45)	0.922 ^a^
	Yes	7	8.8	67.29 (10.58)		74.29 (23.74)	
Activities in the last month. Reflective Writing	No	71	88.8	62.94 (13.99)	0.375 ^a^	74.04 (19.3)	0.565 ^a^
	Yes	9	11.3	67.33 (13.11)		70 (23.5)	
Activities in the last month. Self-care	No	67	83.8	62.19 (13.8)	0.069 ^a^	72.93 (19.81)	0.498 ^a^
	Yes	13	16.3	69.85 (12.95)		77 (19.41)	
Activities in the last month. Voluntary continuing education activities	No	59	73.8	63.47 (13.88)	0.968 ^a^	72.95 (19.62)	0.630 ^a^
	Yes	21	26.3	63.33 (14.23)		75.38 (20.24)	
Activities in the last month. Specialised Retreats	No	71	88.8	62.21 (13.71)	0.026 ^a^	73.9 (19.94)	0.691 ^a^
	Yes	9	11.3	73.11 (11.79)		71.11 (18.44)	
Activities in the last month. Self-care workshops	No	79	98.8	63.46 (13.97)	0.918 ^a^	73.48 (19.79)	0.670 ^a^
	Yes	1	1.3	62 (--)		82 (--)	
Activities in the last month. Team intervention workshops	No	72	90	63.57 (14.55)	0.602 ^c^	72.71 (20.27)	0.233 ^a^
	Yes	8	10	62.25 (5.12)		81.5 (11.29)	
Activities in the last month. Short holidays	No	28	35	60.71 (15.2)	0.200 ^a^	74.5 (20)	0.763 ^a^
	Yes	52	65	64.9 (13.03)		73.1 (19.69)	
Activities in the last month. Meeting with friends	No	26	32.5	65.08 (13.64)	0.467 ^a^	68.23 (23.01)	0.091 ^a^
	Yes	54	67.5	62.65 (14.05)		76.17 (17.52)	
Activities in the last month. Meeting with relatives	No	23	28.8	62.17 (15.73)	0.608 ^a^	78.26 (21.97)	0.179 ^a^
	Yes	57	71.3	63.95 (13.18)		71.7 (18.56)	
Frequency of activities in the last month (*N* = 78)	On a one-off basis.	27	34.6	62.41 (14.72)		73.22 (20.52)	0.637 ^b^
	1 h per week.	6	7.7	56.5 (14.72)	0.009 ^b^	75.5 (16.18)	
	2 h per week.	16	20.5	72 (15.02)		70.25 (23.57)	
	3 h per week.	10	12.8	56.1 (8.96)		71.9 (24.28)	
	4 h per week.	6	7.7	68 (5.62)		72.17 (11.02)	
	5 h per week.	3	3.8	72.67 (1.15)		60.67 (28.67)	
	More than 5 h per week.	10	12.8	55.7 (6.31)		83.5 (5.62)	
Does activities to disconnect from work	Yes	59	73.8	64.64 (15.16)		70.51 (19.8)	0.018 ^a^
	No	21	26.3	60.05 (8.86)	0.101 ^c^	82.24 (16.93)	

^a^ t-Student assuming equality of variances by Levene’s test.; ^b^ ANOVA; ^c^ t-Student without assuming equality of variances by Levene’s test.

**Table 2:** Characteristics of the psycho-occupational health of the sample.

Outcome	Category/Option	*n*	%
Emotional exhaustion	Minimum–Maximun Mean (Standard deviation)		2–53
		23 44	(12.55)
	Low	31	38.8
	Medium	19	23.8
	High	30	37.5
Depersonalisation	Minimum–Maximun Mean (Standard deviation)	0–16	
		2.11	
		(3.25)	
	Low	74	92.5
	Medium	3	3.8
	High	3	3.8
Personal accomplishment	Minimum–Maximun Mean (Standard deviation)	20–56	
		37.3	
		(7.77)	
	Low	30	37.5
	Medium	15	18.8
	High	35	43.8
Maslach Burnout Syndrome	Minimum–Maximun Mean (Standard deviation)	38–97	
		63.44	
		(13.88)	
Confirmed burnout syndrome high emotional exhaustion, high depersonalisation and low self-fulfilment	No	77	96.3
	Yes	3	3.8
Indications of burnout syndrome (at least one of the conditions: high emotional exhaustion, high depersonalisation or low self-fulfilment)	No	34	42.5
	Yes	46	57.5
GS of total satisfaction	Minimum–Maximun Mean (Standard deviation)	29–105	
		73.59	
		(19.69)	
GS of extrinsic satisfaction	Minimum–Maximun Mean (Standard deviation)	17–56	
		38.49	
		(9.96)	
GS of intrinsic satisfaction	Minimum–Maximun Mean (Standard deviation)	12–64	
		34.76	
		(11.3)	

Note: GS: general scale.

**Table 3:** Prevalence of burnout syndrome.

Outcome	Prevalence	95% Confidence Interval
Confirmed Burnout Syndrome ^a^	3.8%	(0.8–10.64%)
Indications of Burnout Syndrome ^b^	57.5%	(45.94–68.49%)

^a^ Met the three conditions: high emotional exhaustion, high depersonalisation and low self-fulfilment; ^b^ meets at least one of the three conditions above.

**Table 4:** Correlation between burnout syndrome and job satisfaction.

		Emotional Exhaustion	Depersonalisation	Personal Accomplishment	r	GS of Extrinsic Satisfaction	GS of Intrinsic Satisfaction
Maslach BS	r	0.843 ^b^	0.414 ^b^	*p*	−0.266 ^a^	−0.321 ^a^	−0.273 ^a^
	*p*	<0.001	<0.001	0.593	0.017	0.004	0.014
Emotional Exhaustion	r		0.488 ^b^	−0.342 ^b^	−0.459 ^b^	−0.482 ^b^	−0.466 ^b^
	*p*		<0.001	0.002	<0.001	<0.001	<0.001
Depersonalisation	r			−0.293 ^b^	−0.303 ^b^	−0.243 ^b^	−0.298 ^b^
	*p*			0.008	0.006	0.030	0.007
Personal accomplishment	r				0.397 ^a^	0.342 ^a^	0.448 ^a^
	*p*				<0.001	0.002	<0.001
GS of Total Satisfaction	r					0.894 ^a^	0.940 ^a^
	*p*					<0.001	<0.001
GS of Extrinsic Satisfaction	r						0.723 ^a^
	*p*						<0.001

Note: GS: general scale; BS: burnout syndrome; r: correlation coefficient; *p*: *p*-value associated to statistical significance. ^a^ Pearson’s correlation coefficient; ^b^ Spearman’s correlation coefficient.

## Data Availability

The data underlying this article cannot be shared publicly to maintain the privacy of individuals that participated in the study. The data will be shared on reasonable request to the corresponding author.

## Supplementary Materials

The following are available online at https://www.mdpi.com/article/10.3390/ijerph18179108/s1, Table S1: socio-demographic characteristics of the sample; Table S2: socio-occupational characteristics of the sample (Part 1) and the relationship with the Maslach’s scores and the level of satisfaction; Table S3: socio-occupational characteristics (Part 2) and the relationship with the Maslach’s scores and the level of satisfaction; Table S4: results of the normality tests.

## References

[1] World Health Organization (2000). Informe Sobre la Salud en el Mundo: 2000: Mejorar el Desempeño de los Sistemas de Salud. https://www.who.int/whr/2000/es/.

[2] Organización Internacional del Trabajo (OIT) (2009). Normas de la OIT Sobre Seguridad y Salud en el Trabajo. https://www.ilo.org/global/standards/subjects-covered-by-international-labour-standards/occupational-safety-and-health/lang--es/index.htm.

[3] Rubio-Jiménez C.J. (2003). Fuentes de Estrés, Síndrome de Burnout y Actitudes Disfuncionales en Orientadores de Instituto de Enseñanza Secundaria. Ph.D. Thesis.

[4] Ley 31/1995, de 8 de Noviembre, de Prevención de Riesgos Laborales. https://www.boe.es/buscar/act.php?id=BOE-A-1995-24292.

[5] Real Decreto 39/1997, de 17 de Enero, Por el que se Aprueba el Reglamento de los Servicios de Prevención. https://www.boe.es/buscar/act.php?id=BOE-A-1997-1853.

[6] Maslach C., Schaufeli W.B., Leiter M.P. (2001). Job Burnout. Annu. Rev. Psychol..

[7] Cheung C.-K., Chow E.O.-W. (2011). Reciprocal influences between SB and effectiveness in professional care for elders. Soc. Work Health Care.

[8] Maslach C., Schaufeli W., Maslach C., Marek T. (1993). Burnout: A Multidimensional Perspective. Professional Burnout: Recent Developments in Theory and Research.

[9] Kearney M., Weininger R.B., Vachon M.L., Harrison R.L., Monut B.M. (2009). Self-care of physicians caring for patients at the end of life. JAMA.

[10] Escribà V., Artazcoz L., Pérez S. (2008). Efecto del ambiente psicosocial y de la satisfacción laboral en el síndrome de SB en médicos especialistas. Gac. Sanit..

[11] González P., Peiró J.M., Bravo M.J., Peiró J.M., Prieto F. (1996). Calidad de vida laboral. Tratado de Psicología del Trabajo. Vol.II: Aspectos Psicosociales del Trabajo.

[12] Smart D., English A., James J., Wilson M., Daratha K.B., Childers B., Magera C. (2014). Compassion fatigue and satisfaction: A cross-sectional survey among US healthcare workers. Nurs. Health Sci..

[13] Sequera M., Miranda C., Massegú C., Pablos C., González A. (2015). Musicoterapia en la demencia del paciente anciano: Fundamentos, aplicaciones y evidencia científica actual. Psicogeriatría.

[14] EMTC (Confederación Europea de Musicoterapia) Training Programs in Spain c2019. https://www.emtc-eu.com/spain.

[15] AEMP (Asociación Española de Musicoterapeutas Profesionales) Documentos Técnicos. https://musicoterapeutas.wixsite.com/aemp/documentos.

[16] Bruscia K.E. (2006). Musicoterapia.

[17] Federación Española de Asociaciones de Musicoterapia. http://feamt.es/.

[18] Oppenheim L. (1984). A work values profile of university music therapy and performance majors. J. Music Ther..

[19] Kim Y. (2012). Music Therapists’ Job Satisfaction, Collective Self-Esteem, and SB. Arts Psychother..

[20] Chang K. (2014). An Opportunity for Positive Change and Growth: Music Therapists’ Experiences of burnout. Can. J. Music Ther..

[21] Hills B., Ian N., Lucy F. (2000). A study of burnout and multidisciplinary team-working amongst professional music therapists. Br. J. Music Ther..

[22] Carvallo L., Ilariucci L. (2013). Índices de Estrés laboral y la Prevalencia del SB, en Profesionales Musicoterapeutas que Trabajan en la Provincia de Bs. As. Tesis de Licenciatura.

[23] Boluarte A. (2014). Propiedades psicométricas de la Escala de satisfacción laboral de Warr, Cook y Wall, versión en español. Rev. Méd. Hered..

[24] Warr P., Cook J., Wall T. (1979). Scales for the measurement of some work attitudes and aspects of psychological well-being. J. Occup. Psychol..

[25] Stewart D. (2000). The state of the UK music therapy profession: Personal qualities, working models, support networks and job satisfaction. Br. J. Music Ther..

[26] Gooding L.F. (2018). Work-life factors and job satisfaction among music therapy educators: A national survey. Music Ther. Perspect..

[27] Gooding L.F. (2019). Burnout among music therapists: An integrative review. Nord. J. Music Ther..

[28] Decuir A.A., Policastro-Vega V. (2010). Career longevity: A survey of experienced professional music therapists. Arts Psychother..

[29] Braswell C., Decuir A., Jacobs K. (1989). Job satisfaction among music therapists. J. Music Ther..

[30] Dos Santos E., Alves A., de Oliveira C.R., Esperidâo E. (2015). Cuidando de quem cuida: Uma revisão integrativa sobre a musicoterapia como possibilidade terapêutica no cuidado ao cuidador. Rev. Música Hodie.

[31] Taylor D. (1997). Biomedical Foundations of Music as Therapy.

[32] Weinert A.B. (1985). Manual de Psicología de la Organización.

[33] Robbins S.P. (1987). Comportamiento Organizacional.

[34] Robbins S.P., Coulter M. (1996). Administración.

[35] Maslach C., Jackson S., Leiter MPMaslach S.B. (1996). Inventory Manual.

[36] Gil P.R. (2002). Validez factorial de la adaptación al español del Maslach Burnout Inventory-General Survey. Salud Pública de Méx..

[37] Segurado A., Agulló E. (2002). Calidad de vida laboral: Hacia un enfoque integrador desde la Psicología Social. Psicothema.

[38] Fowler K.L. (2006). The relation between personality characteristic, work environement and the professional well-being of music therapist. J. Music Ther. Fall.

[39] Vega V.P. (2010). Personality, burnout and longevity among professional music therapist. J. Music Ther..

[40] Sabbatella P.L., Mercadal M. (2014). Perfil profesional y laboral de los musicoterapeutas españoles: Un estudio descriptivo. Rev. Bras. Musicoter..

[41] Méndez-Campos R. (2015). Estudio Sobre la Prevalencia de la Fatiga de la Compasión y su Relación con el Síndrome de Burnout en Profesionales de Centros de Mayores en Extremadura. Ph.D. Thesis.

[42] Cifre E., Llorens S. (2001). Burnout en Profesores de la UJI: Un estudio diferencial.

[43] Galicia F.A., González-Zermeño M.A. (2009). Estrés, agotamiento profesional (burnout) y salud en profesores de acuerdo a su tipo de contrato. Cien Trab..

[44] Ortiz T., Birriel J., Ortega R. (2004). Género, profesiones sanitarias y salud pública. Gac. Sanit..

[45] Butler S.K., Madonna G.G. (2005). Collective self-esteem and burnout in professional school counselors. Prof. Sch. Couns..

[46] Rainey H.G. (2014). Understanding and Managing Public Organizations.

[47] Berry S. (2017). An analysis of SB and Music Therapy Methodologies. Ph.D. Thesis.

[48] Yu K., Sang L., Sang L. (2007). Counselors’ collective self-esteem mediates job dissatisfaction and client relationships. J. Employ. Couns..

[49] Register D. (2013). Professional recognition of music therapy: Past, present, and future. Music Ther. Perspect..

[50] Letulė N., Ala-Ruona E. (2016). An overview of the music therapy professional recognition in the EU. Spec. Ugdym..

[51] Ridder H.M., Adrienne L., Suvini F. (2015). The role of the EMTC for development and recognition of the music therapy profession. Approaches Music Ther. Spec. Music Educ..

