# Supplementary material for: Prevalence of Burnout Syndrome and Job Satisfaction in Music Therapists in Spain: A Cross-Sectional, Descriptive Study
Source: Int J Environ Res Public Health. 2021 Aug 29;18(17):9108. doi: 10.3390/ijerph18179108 (PMC8430565; doi:10.3390/ijerph18179108)
Supplement: Supplementary file 1 [file ijerph-18-09108-s001.zip › ijerph-1309473-supplementary.pdf]

SUPPLEMENTAL MATERIAL

Supplemental Table S1. Socio-demographic characteristics of the sample.

Outcome	Category	n	%
**Age**	20-29	14	17.5
	30-39	31	38.8
	40-49	23	28.8
	50-59	11	13.8
	Over 60	1	1.3
**Gender**	Male	12	15
	Female	68	85
**Educational level**	Music Therapy Masters Degree (60ECTS)	65	81.3
	Music Therapy Degree (Foreign country)	3	3.8
	Others	12	15
**Marital status**	Single	18	22.5
	With stable partner	41	51.3
	Without stable partner	1	1.3
	Married or civil partner	19	23.8
	Widowed (less that 2 years ago)	1	1.3
**Region of employment**	Andalucía	8	10
	Aragón	7	8.8
	Asturias	4	5
	Castilla La Mancha	3	3.8
	Castilla and León	3	3.8
	Ceuta	1	1.3
	Extremadura	14	17.5
	Galicia	2	2.5
	Balearic Islands	10	12.5
	Canary Islands	4	5
	La Rioja	1	1.3
	Madrid	12	15
	Murcia	1	1.3
	Basque Country	1	1.3
	Valencia	9	11.3

Supplemental Table S2. Socio-occupational characteristics of the sample (part 1) and the relationship with the Maslach's scores and the level of satisfaction.

Outcome.	Category/option	n	%	Maslach Mean Score (SD)	p-value	Mean Job Satisfaction (SD)	p-value
**Combines working as a music therapist with other jobs.**	Yes	61	76.3	62.23 (13.79)		73.74(20.36)	
	No	19	23.8	67.31 (13.81)	0.165[a]	73.11 (17.86)	0.904[a]
**Duties performed at the workplace**	Care duties	48	60	62.15 (13.06)		70.63 (18.75)	
	Administration and management	5	6.3	61 (12.1)	0.449[b]	67.6 (17.95)	0.111[b]
	Care duties and Administration/ management	27	33.8	66.19 (15.58)		79.96 (20.67)	
**Type of contract**	Fixed term contract	13	16.3	54.38 (10.14)		77 (18.76)	
	Indefinite contract	20	25	67.45 (13.98)		69.35 (22.24)	
	Short-term contract	6	7.5	63.17 (10.01)	**0.001[b]**	62.17 (23.22)	**0.041[b]**
	Trainee contract	3	3.8	90 (8.89)		55 (13.23)	
	For specific work and services	6	7.5	59.67 (9.83)		64.33 (17.91)	
	Self-employed	32	40	62.88 (13.36)		80.47 (16.05)	
**Length of service in the profession**	Less than 1 year	13	16.3	69.46 (18.26)		68.69 (19.67)	
	1-4 years	37	46.3	59.43 (13.49)		70.19 (19.04)	
	5-9 years	14	17.5	69.79 (11.31)	0.067[b]	77.79 (20.3)	0.258[b]
	10-19 years	11	13.8	62.64 (8.54)		81.18 (22.59)	
	20-29 years	5	6.3	61.4 (12.48)		83 (10)	
**Length of time in current position**	Less than 1 year	12	15	67.17 (16.97)		67.25 (23.21)	
	1-4 years	38	47.5	61.92 (12.51)		68.97 (18.6)	
	5-9 years	11	13.8	66.82 (14.78)	0.651[b]	80.55 (14.28)	0.070[b]
	10-19 years	17	21.3	63.09 (14.62)		81.18 (19.34)	
	20-29 years	1	1.3	62 (--)		94 (--)	
	Over 30 years	1	1.3	47 (--)		99 (--)	
**Working hours**	Less than 5 hours a week	26	32.5	65.69 (15.5)		64 (20.35)	
	Between 5 and 10 hours a week	15	18.8	58.27 (14.39)		74.87 (26.44)	

	Between 10 and 20 hours a week	18	22.5	65.28 (14.62)	0.377b	74.83 (13.5)	**0.016b**
	Between 20 and 40 hours a week	15	18.8	64.8 (10.73)		82.73 (10.53)	
	Over 40 hours a week	6	7.5	57.67 (7.06)		85.33 (16.17)	
**Working shift**	Morning	20	25	57.45 (14.59)		70.55 (19.78)	
	Afternoon-evening	29	36.3	69.07 (14.78)		68.79 (20.91)	
	Night	1	1.3	50 (--)		86 (--)	0.234b
	Rotating shift, morning-afternoon on week days.	16	20	62.75 (12.11)		78.19 (18.73)	
	Rotating shift, morning-afternoon-evening-nights on week days.	1	1.3	52 (--)	0.071b	102 (--)	
	Rotating shift, morning-afternoon on weekends or bank holidays.	11	13.8	64.64 (8.19)		82.55 (16.01)	
	Rotating shift, morning-afternoon-evening-nights on weekends and bank holidays.	2	2.5	53 (0)		67 (0)	

Note: SD: Standard Deviation.

Supplemental Table S3. Socio-occupational characteristics (part 2) and the relationship with the Maslach's scores and the level of satisfaction.

Outcome	Category/option	n	%	Maslach Mean Score (SD)	p-value	Mean Job Satisfaction (SD)	p-value
**Provides training**	Yes	40	50	67 (13.57)		75.53 (18.76)	
	No	40	50	59.88 (13.42)	**0.021[a]**	71.65 (20.62)	0.382[a]
**Working in antinatal care**	No	73	91.3	63.73 (14.16)		72.79 (19.59)	
	Yes	7	8.8	60.43 (10.91)	0.552[a]	81.86 (20.29)	0.247[a]
**Working in premature paediatric intensive care units**	No	75	93.8	62.99 (14.13)	0.263[a]	73.17 (20.22)	
	Yes	5	6.3	70.2 (7.05)		79.8 (6.38)	0.100[c]
**Working in early stimulation**	No	67	83.8	63.36 (14.46)		73.24 (19.41)	
	Yes	13	16.3	63.85 (10.89)	0.909[a]	75.38 (21.78)	0.722[a]
**Working in early care intervention**	No	66	82.5	64.35 (14.48)		72.26 (19.64)	
	Yes	14	17.5	59.14 (9.9)	0.115[c]	79.86 (19.37)	0.191[a]
**Working in the field of disability**	No	44	55	63.66 (14.92)		68.66 (21.37)	
	Yes	36	45	63.17 (12.7)	0.876[a]	79.61 (15.67)	**0.010[c]**
**Working in the field of education**	No	41	51.3	62.29 (14.36)	0.453[a]	79.37 (18.14)	
	Yes	39	48.8	64.64 (13.44)		67.51 (19.63)	**0.006[a]**
**Working in the field of mental health**	No	68	85	62.51 (14.19)	0.158[a]	71.49 (19.79)	
	Yes	12	15	68.67 (11.09)		85.5 (14.72)	**0.022[a]**
**Working in a hospital setting**	No	69	86.3	63.7 (13.91)	0.680[a]	79.99 (20.19)	
	Yes	11	13.8	61.82 (14.26)		83.64 (12.67)	0.068[a]
**Working in a community setting**	No	75	93.8	63.2 (14.18)	0.557[a]	73.89 (19.35)	
	Yes	5	6.3	67 (8.28)		69 (26.45)	0.594[a]
**Working in the field of geriatrics**	No	62	77.5	63.02 (13.59)	0.617[a]	72.65 (21.31)	
	Yes	18	22.5	64.89 (15.16)		76.83 (12.51)	0.301[c]
**Working in the field of neurorehabilitation**	No	62	77.5	63.81 (14.41)	0.662[a]	73.98 (20.34)	
	Yes	18	22.5	62.17 (12.15)		72.28 (17.7)	0.751[a]
	No	76	95	63.49 (14.16)	0.891[a]	74.36 (19.4)	

**Working in the field of Fibromyalgia and chronic pain**	Yes	4	5	62.5 (7.94)		59 (22.32)	0.129[a]
**Working in the field of palliative care**	No	74	92.5	62.65 (13.48)	0.074[a]	72.97 (19.71)	
	Yes	6	7.5	73.17 (16.41)		81.17 (19.43)	0.330[a]
**Type of working centre**	Public	26	32.5	66.92 (17.54)		68.23 (20.86)	
	Private	50	62.5	62.26 (11.65)	0.193[b]	76.06 (19.4)	
	Non governmental organisation	4	5	55.5 (9)		77.5 (7.68)	
**Combines music therapy with other jobs**	Yes, regularly	44	55	64.84 (15.45)		70.02 (21.38)	
	Yes, ocasionally	21	26.3	63.9 (13.74)	0.330[b]	71.48 (17.46)	
	No	15	18.8	58.67 (7.35)		87 (10.39)	
**Intention to leave the job**	Yes	20	25	67.6 (14.87)	0.122[a]	58.6 (17.8)	**<0.001[a]**
	No	60	75	62.05 (13.38)		78.58 (17.76)	
**Maintains the enthusiasm for working in the current position**	Yes	38	47.5	63.08 (14.68)	0.116[b]	73.92 (19.84)	
	No, it has increased	18	22.5	58.67 (12.79)		87.39 (16.17)	**<0.001[b]**
	No, it has decreased	24	30	67.58 (12.57)		62.71 (15.22)	
**Receives training**	Yes	78	97.5	63.01 (13.77)		73.91 (19.19)	0.363[a]
	No	2	2.5	80 (7.07)	0.088[a]	61 (43.84)	
**Number of patients treated individually per week**	0-5	56	70	60.68 (13.37)		71.59 (18.85)	0.097[b]
	6-10	17	21.3	74.59 (12.17)	**0.001[b]**	74 (23.05)	
	Over 10	7	8.8	58.43 (7.16)		88.57 (11.37)	
**Number of patients treated in groups per week**	0-10	43	53.8	63.91 (15.27)		73.51 (21.8)	0.975[b]
	11-20	15	18.8	64.47 (12.89)	0.810[b]	72.8 (22.18)	
	Over 20	22	27.5	61.82 (11.99)		74.27 (13.36)	
**Feels valued by colleagues**	Yes	77	96.3	63.6 (13.97)	0.605[a]	74.14 (19.86)	
	No	3	3.8	59.33 (13.05)		59.33 (2.31)	**<0.001[c]**
**Feels valued by the centre's director (n=79)**	Yes	64	81	62.47 (13.87)	0.221[a]	77.55 (17.76)	
	No	15	19	67.4 (14.13)		54.6 (15.45)	**<0.001[a]**

	Yes	15	18,8	71.27 (19.84)	0.046[b]	68.33 (25.03)	0.234[b]
**Often works with a therapeutic colleague**	No	44	55	62.18 (11,86)		72.66 (19.93)	
	Sometimes	21	26.3	(60.48 (11,12)		79.29 (13.48)	
**Taking medically prescribed psychotropic medication (n=79)**	Yes	11	13.9	57.55 (8,09)	0.034[c]	63.91 (21.41)	0.074[a]
	NO	68	86.1	64.34 (14.52)		75.35 (19.16)	

[a]t-Student assuming equality of variances by Levene's test.; [b]ANOVA ;[c]t-Student without assuming equality of variances by Levene's test.

Supplemental Table S4. Results of the normality tests.

	**Z de Kolmogorov-Smirnov**	**p-value**	**Normal**
MBI	0.916	0.372	Yes
MBI Emotional exhaustion	1.571	0.014	No
MBI Depersonalisation	2.308	0.000	No
MBI Personal accomplishment	1.139	0.150	Yes
GS of Total satisfaction	1.030	0.239	Yes
GS of Extrinsic satisfaction	1.208	0.108	Yes
GS of Intrinsic satisfaction	0.980	0.292	Yes

Note: BS: Burnout Syndrome; MBI: Maslach Burnout Inventory; GS: General Scale.
